# Enhancing the Ground Truth Disparity by MAP Estimation for Developing a Neural-Net Based Stereoscopic Camera

**DOI:** 10.3390/s24237761

**Published:** 2024-12-04

**Authors:** Hanbit Gil, Sehyun Ryu, Sungmin Woo

**Affiliations:** Department of Information and Communication Engineering, Korea University of Technology and Education (KOREATECH), Cheonan-si 31253, Republic of Korea; hb3568@koreatech.ac.kr (H.G.); ryurtrt@koreatech.ac.kr (S.R.)

**Keywords:** stereo vision, deep learning, disparity map, MAP estimation, Semi-Global Matching, neural network, interpolation, autonomous driving

## Abstract

This paper presents a novel method to enhance ground truth disparity maps generated by Semi-Global Matching (SGM) using Maximum a Posteriori (MAP) estimation. SGM, while not producing visually appealing outputs like neural networks, offers high disparity accuracy in valid regions and avoids the generalization issues often encountered with neural network-based disparity estimation. However, SGM struggles with occlusions and textureless areas, leading to invalid disparity values. Our approach, though relatively simple, mitigates these issues by interpolating invalid pixels using surrounding disparity information and Bayesian inference, improving both the visual quality of disparity maps and their usability for training neural network-based commercial depth-sensing devices. Experimental results validate that our enhanced disparity maps preserve SGM’s accuracy in valid regions while improving the overall performance of neural networks on both synthetic and real-world datasets. This method provides a robust framework for advanced stereoscopic camera systems, particularly in autonomous applications.

## 1. Introduction

Depth estimation through stereo vision using deep learning is extensively applied in fields like autonomous driving, robotics, and augmented reality [1,2,3]. The accuracy of this technology hinges on high-quality ground truth data [4]. Although synthetic datasets can produce perfect ground truth depth maps, their use in commercial camera manufacturing is limited due to the differences between synthetic and real-world images, such as variations in lighting conditions, color changes from multiple light sources, and light reflections and transmissions [5,6].

To mitigate these issues, ground truth disparity maps are usually created using traditional stereo-matching methods like Semi-Global Matching (SGM) [7]. SGM is a dependable and precise method that calculates actual distances based on camera calibration parameters [8]. It determines disparity by minimizing the matching cost across multiple scan lines, considering pixel differences in the context of their surrounding regions in both the left and right images. However, SGM encounters difficulties when there is significant occlusion, especially with foreground objects, and in regions with flat surfaces that lack texture, making it hard to accurately compute binocular disparity. In such instances, deeming the disparity values as “invalid” ensures higher confidence in the normal values.

Intel’s D-series (Intel Corporation, Santa Clara, CA, USA), one of the popular off-the-shelf stereoscopic cameras, employs 720 p/30 fps real-time circuitry to use the SGM method to generate depth information. The accuracy of distance estimation with this camera is reported to be within two percent of the actual distance, which is accepted for the autonomous driving application of distance measurement. Additionally, Intel’s D-series uses infrared (IR) projection to create patterns that help calculate disparity, minimizing ambiguity in textureless regions [9,10]. Post-processing techniques, such as edge-preserving filtering [11], spatial hole-filling [12], and temporal filtering [13] further improve the disparity map generated by SGM.

However, these post-processing methods are still insufficient in addressing the fundamental challenges of achieving better disparity maps when large invalid areas occur due to severe occlusion or lack of texture in the scenes. Above all, deep learning-based methods exhibit poor generalization when applied to the real-world datasets lacking ground truth. In this study, we propose an interpolation method to enhance SGM disparity maps, enabling their use as ground truth data for training lightweight neural network models designed for disparity estimation in mobile stereoscopic cameras. To validate this, we designed a neural network and its variants specifically tailored for disparity estimation from stereo images and trained the models on a dataset of over 4000 stereo image pairs captured using a custom-designed stereo camera equipped with IR projectors. The experimental results with synthetic and real-world datasets demonstrate that the improved ground truth significantly enhances the network’s output, resulting in sharper and more visually accurate disparity maps.

## 2. Related Work

Image interpolation methods primarily include Bilinear Interpolation and Spline Interpolation. Bilinear Interpolation utilizes the values of four neighboring pixels for interpolation, making it easy to implement and computationally efficient. This method is beneficial for real-time processing but struggles to restore fine details. Spline Interpolation, on the other hand, uses a higher-dimensional polynomial spline function to achieve smoother interpolation results, albeit with increased computational costs. It is particularly effective for continuous or smoothly varying data. Partial Differential Equation (PDE)-based inpainting methods restore damaged image regions by propagating surrounding pixel information using mathematical techniques. These approaches preserve edge continuity and important structures through isotropic or anisotropic diffusion, ensuring smooth transitions between damaged and undamaged areas.

Among various specialized interpolation methods for stereo matching, an iterative color-depth Minimum Spanning Tree (MST) cost aggregation algorithm was introduced to improve accuracy in textureless regions [14]. A joint matching cost method was also developed by combining the Sum of Absolute Differences (SAD) and Census transform, enhancing stereo matching accuracy [15]. Additionally, a local stereo matching approach was proposed that integrates an adaptive exponentially weighted moving average (EWMA) filter with Simple Linear Iterative Clustering (SLIC) segmentation, improving both computational efficiency and accuracy [16]. To address occlusion challenges in disparity estimation for dynamic scenes, a local stereo matching method was proposed, combining support weights with motion flow [17].

Several segmentation-based stereo matching techniques [18,19,20] have also demonstrated effectiveness in handling occluded regions and object boundaries. Additionally, a multi-step disparity refinement framework was introduced to classify outliers and prevent error propagation [21]. An unsupervised stereo matching network has also been proposed, incorporating occlusion handling through a ternary classification system and directed disparity smoothing loss [22]. For textureless regions in stereo images, plane-fitting techniques [23] and non-local cost aggregation methods [24] have been suggested. Geodesic filters were applied to enhance real-time performance and improve disparity accuracy [25,26]. Lastly, belief propagation-based optimization methods were introduced to enhance disparity accuracy, particularly in low-texture areas and occlusion boundaries [27,28].

Disparity refinement methods encompass a range of approaches beyond occlusion handling and interpolation. Techniques using Markov Random Fields (MRF) [29,30,31] have been widely applied, along with edge-preserving filters [32] box filters [32], fast-weighted median filters [33], and multi-step methods incorporating voting strategies [34,35,36,37,38,39]. Additionally, various approaches [40,41,42,43,44] have been proposed to enable real-time stereo matching for FPGA or GPU implementation.

One of the most significant advances in stereo matching has been the adoption of deep learning-based methods. A unified deep learning architecture was proposed to enhance stereo confidence estimation by jointly utilizing the matching cost volume and disparity map, thereby improving stereo matching robustness [45]. For light field (LF) depth estimation, a method was introduced to learn the distribution of subpixel disparities [46]. A CNN was also employed to compute patch similarity, achieving speed and accuracy improvements in stereo matching [47]. Additionally, an unsupervised learning framework for optical flow estimation explicitly modeled occlusions to improve performance in challenging conditions [48]. The use of 3D feature volumes in intermediate layers of deep neural networks has further demonstrated effectiveness in accurate disparity calculation [49,50,51,52]. More recently, iterative stereo matching techniques have shown outstanding performance in preserving edges and shapes [53,54].

Deep learning-based interpolation methods enable broad inpainting by learning inferred structural details through neural networks, allowing models to reconstruct cohesive and contextually accurate regions within images. A Generative Multi-column Convolutional Neural Network (GMCNN) was proposed for image inpainting to restore missing regions by propagating context-derived information from surrounding areas [55]. For translation-variant interpolation (TVI) tasks such as image inpainting and super-resolution, a Shepard Convolutional Neural Network (ShCNN) was introduced to propagate information from known to unknown regions through spatially variant pixel weighting [56]. Chen et al. [57] introduced a pluralistic image inpainting approach leveraging latent codes and a bidirectional transformer to effectively restore large masked regions. A mask-aware inpainting framework was also developed to improve feature learning for missing regions [58], while a transformer-based model targeted large-hole inpainting [59]. Recent inpainting approaches have increasingly leveraged diffusion models for enhanced outcomes [60,61,62,63,64].

Despite the advantages of deep learning-based stereo matching techniques in achieving natural edge estimation and object description in disparity maps, they are computationally intensive and thus less suited for real-time stereo camera applications. Moreover, generating accurate real-world training data for precise distance estimation remains challenging, as inpainting methods, while visually plausible, often lack accuracy in estimating distances. Furthermore, recent deep learning-based approaches frequently suffer from poor generalization, especially when applied to diverse real-world scenarios. To address these issues, we propose a Maximum a Posteriori (MAP) estimation-based method to enhance disparity maps generated by Semi-Global Matching (SGM), making them more suitable as high-quality training data for lightweight neural networks in real-time stereoscopic camera applications. Our MAP-based interpolation approach not only improves edge consistency and disparity accuracy but also provides a robust foundation for disparity estimation in autonomous and real-time scenarios. The main contributions of our work are as follows:

1. **MAP-Based Interpolation with Enhanced Likelihood Calculation for Invalid Disparity Pixels**: Our MAP estimation approach leverages surrounding disparity information as the prior and utilizes cosine similarity as the likelihood, complemented by a preprocessing step that standardizes pixel intensity and applies masking. This posterior probability estimation identifies the most plausible disparity values for invalid regions caused by occlusions, textureless surfaces, and inconsistencies between left and right images in stereo vision.
2. **Practical Validation in Real-World Scenarios Where Ground Truth Is Difficult To Obtain**: The proposed method demonstrates robustness and applicability in both ground truth (GT)-available and GT-unavailable scenarios. In environments without GT, where accurate disparity maps are inaccessible, learning-based methods trained on different datasets often struggle with challenges such as indistinct boundaries and the propagation of incorrect predictions when applied to unseen images. Our approach overcomes these limitations by employing MAP-based interpolation to generate sharper and more reliable disparity maps. Evaluations conducted on a comprehensive dataset of over 4000 real-world stereo images validate the proposed method’s ability to generalize effectively across diverse environments, emphasizing its practical usability in real-world applications where GT is unavailable.
3. **Improved Ground Truth for Lightweight Neural Network Training**: We evaluate the impact of our enhanced ground truth data on various lightweight neural network architectures optimized for mobile applications. Specifically, we compare network performance using original SGM-based ground truth data against ground truth data enhanced by our proposed method. Results indicate that the proposed enhancement significantly improves output quality and generalization of these networks, demonstrating its value for real-time stereoscopic applications with limited computational resources.

## 3. Proposed MAP Estimation

**Figure 1:**
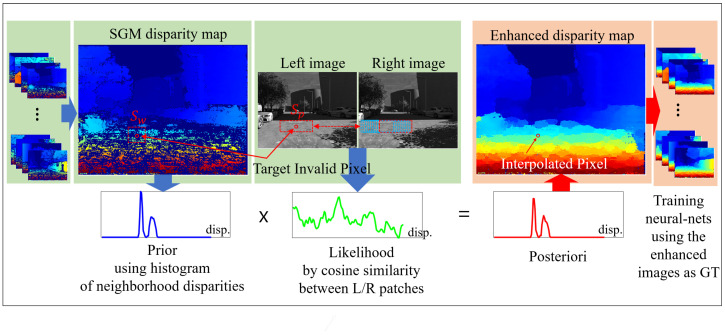
The proposed framework for enhancing SGM disparity map.

Figure 1 outlines the proposed framework for improving the SGM disparity map by filling in invalid pixels. The process begins with an SGM disparity map that contains missing regions. A prior is calculated using neighboring disparities within a fixed window, while the likelihood is determined through cosine similarity between patches from the left and right images. These prior and likelihood calculations are combined to create a posterior distribution, which estimates the disparities for the invalid pixels. The result is a refined disparity map with interpolated pixels, enhancing the accuracy of the original SGM disparity map.

### 3.1. Invalid Regions of SGM

**Figure 2:**
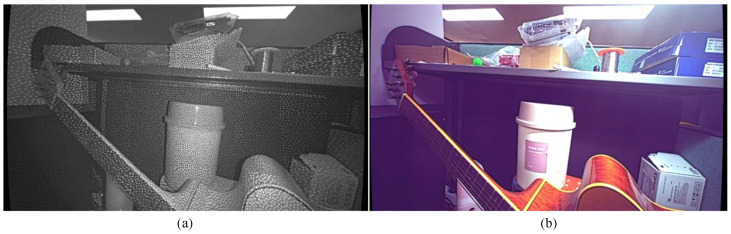
Example of left (**a**) and RGB (**b**) images.

**Figure 3:**
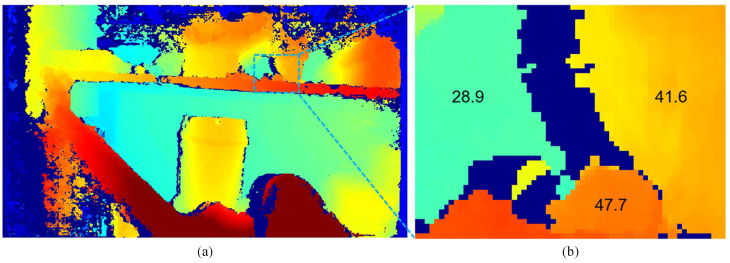
(**a**) Disparity map generated by SGM for the images in Figure 1. (**b**) Enlarged view of (**a**). Dark blue pixels indicate “invalid” regions. The numbers shown represent disparity values for each grouped region.

In SGM, invalid pixels occur when matches cannot be found, often due to occlusions, similar costs in neighboring patches, or disparities between left and right images. Figure 2a,b show the left and RGB images, both captured by a stereo camera system aligned with an IR projector. In Figure 2b, the RGB camera’s color filter blocks infrared wavelengths, rendering the IR patterns invisible. However, in Figure 2a, the irregular patterns projected by the IR aid in stereo matching by adding texture to otherwise featureless areas, leading to a denser disparity map. Despite this, some invalid pixels caused by occlusion remain unavoidable. These occlusions are a result of object and camera geometry, where closer objects and greater distance between cameras increase occlusion effects. Figure 3a shows the SGM disparity map created from the left and right images in Figure 2, with a “jet” colormap used to represent closer objects in red and farther objects in blue. Invalid pixels appear as dark blue. Figure 3b provides a magnified view of the red box in Figure 3a, with numbers representing approximate average disparity values for different regions. In this figure, the disparity map is aligned with the left camera as the reference.

### 3.2. Prior Probability of Invalid Pixels

**Figure 4:**
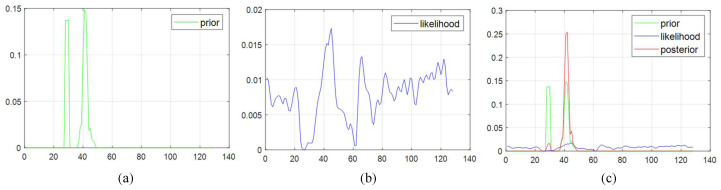
(**a**) Prior probability, (**b**) Likelihood, and (**c**) Posterior distribution of an invalid pixel from Figure 3.

To estimate the empty disparities in SGM using MAP, a prior probability P(θ) is established. θ represents the disparity of an invalid pixel. The key idea in setting the prior probability is that the occluded disparity is limited to the disparities surrounding the target. To do this, a histogram of the valid pixels within a $S_{w}$-sized window centered by a target pixel is constructed in the range of zero to maximum disparity, and then the histogram is normalized so that they can be represented as probabilities. In the resulting normalized histogram, the probabilities tend to be concentrated in distinct disparity spots, leading to zero probability for adjacent high disparity priors, which is unlikely. To address this, a 1D convolution is applied to spread out the initial prior distribution. Figure 4a illustrates the resulting prior distribution of the target pixel from Figure 3b with $S_{w}$ set to be 17×17. The horizontal axis represents disparity, and the maximum disparity is set to 128. The convolution does not affect the distribution in the range of 50 to the maximum disparity, indicating that regardless of the likelihood values given later, the posterior will be limited to the non-zero range.

### 3.3. Likelihood Probability

**Figure 5:**
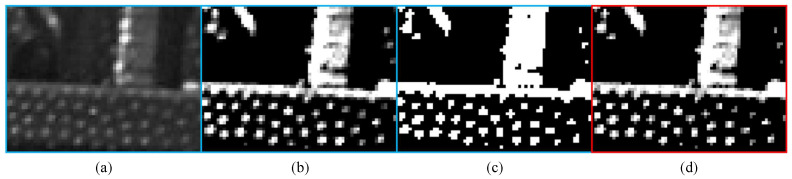
Preprocessing steps for the proposed method: (**a**) Original cropped patch, (**b**) Standardized patch, (**c**) Mask, and (**d**) Masked patch.

To determine the likelihood of invalid pixels P(D|θ), a $S_{p}$-sized patch centered on the target pixel in the left image is compared with corresponding moving cropped regions of the same size in the right image. The similarity between the left and right patches is measured using cosine similarity. In our case, the brightness of the captured image has slight differences between pairs. Thus, the following preprocessing steps are initially performed on the left and right patches to facilitate image comparison:

1. The original captured image is standardized as follows:
  (1) $$x_{st}=\frac{x_{ori}-\mu }{\sigma }$$
  where μ and σ are the average and standard deviation of the patch, respectively.
2. The standardized image $x_{st}$ is then masked out so that the pixels with low intensity are not regarded as distinct features:
  (2) $$\tilde{x}=\left\{\begin{array}{cc} x_{st} & \mathrm{if}\;x_{st}>I_{th},\\ 0 & \mathrm{otherwise} \end{array}\right.$$
  where $I_{th}$ is the intensity threshold, set to −0.7 throughout the study. Figure 5 illustrates the above preprocessing step, consecutively. Therefore, in the preprocessing step, only the pixels with relatively high intensities are used for comparison.

**Figure 6:**
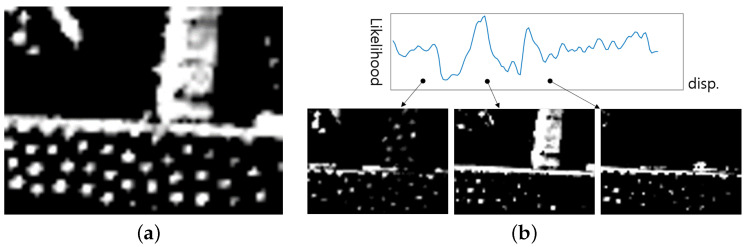
(**a**) Left masked cropped patch. (**b**) Right cropped candidate patches.

Figure 6 illustrates the left patch and some of the corresponding candidates patches from the right image, used to estimate the invalid pixels. The initial likelihood is calculated using the cosine similarity between the flattened left ${\tilde{x}}^{L}$ and *i*-th horizontal displacement image ${\tilde{x}}_{i}^{R}$ as follows:

(3) $$\cos\left({\tilde{x}}^{L},{\tilde{x}}_{i}^{R}\right)=\frac{{\tilde{x}}^{L}\cdot {\tilde{x}}_{i}^{R}}{∥{\tilde{x}}^{L}∥\;∥{\tilde{x}}_{i}^{R}∥}$$

After normalizing the cosine similarities over the range from one to the maximum disparity, the likelihood probability distribution P(D|θ) is obtained as illustrated in Figure 4b with $S_{p}$ set to a 48×8 window. However, cosine similarity is sensitive to noise and geometrical displacement, leading to a probability distribution that is not smooth.

### 3.4. Posterior Probability

The posterior probability of the invalid pixels P(θ|D) is determined by Bayes’ rule and the estimated disparity ${\hat{\theta }}_{\mathrm{MAP}}$ by the MAP estimation are determined as follows:

(4) $${\hat{\theta }}_{\mathrm{MAP}}=\underset{\theta }{\mathrm{argmax}}\;P\left(D|\theta \right)P\left(\theta \right)$$

Figure 4c illustrates the posterior probability obtained through MAP estimation using the prior from Figure 4a and the likelihood from Figure 4b. Since the posterior probability is the product of the prior probability and the likelihood, any values with a prior probability of zero will also have a posterior probability of zero. Although considering only the likelihood might suggest certain disparity values, combining it with the prior probability, which takes into account the disparity values near the invalid region, results in a more accurate estimation of the optimal disparity value. In the example shown in Figure 3, the disparity of the invalid region is estimated to be 47.7, the value with the highest posterior probability among those with a non-zero prior probability.

## 4. Experimental Result

The performance of the proposed method is evaluated on both synthetic datasets with GT available and real-world datasets where GT is unavailable. Specifically, for the real-world dataset we collected, we demonstrate how the disparity map enhanced by the proposed method improves the output of each lightweight neural network capable of running in real-time on mobile devices.

### 4.1. Performance Evaluation on Synthetic Datasets with Ground Truth

**Figure 7:**
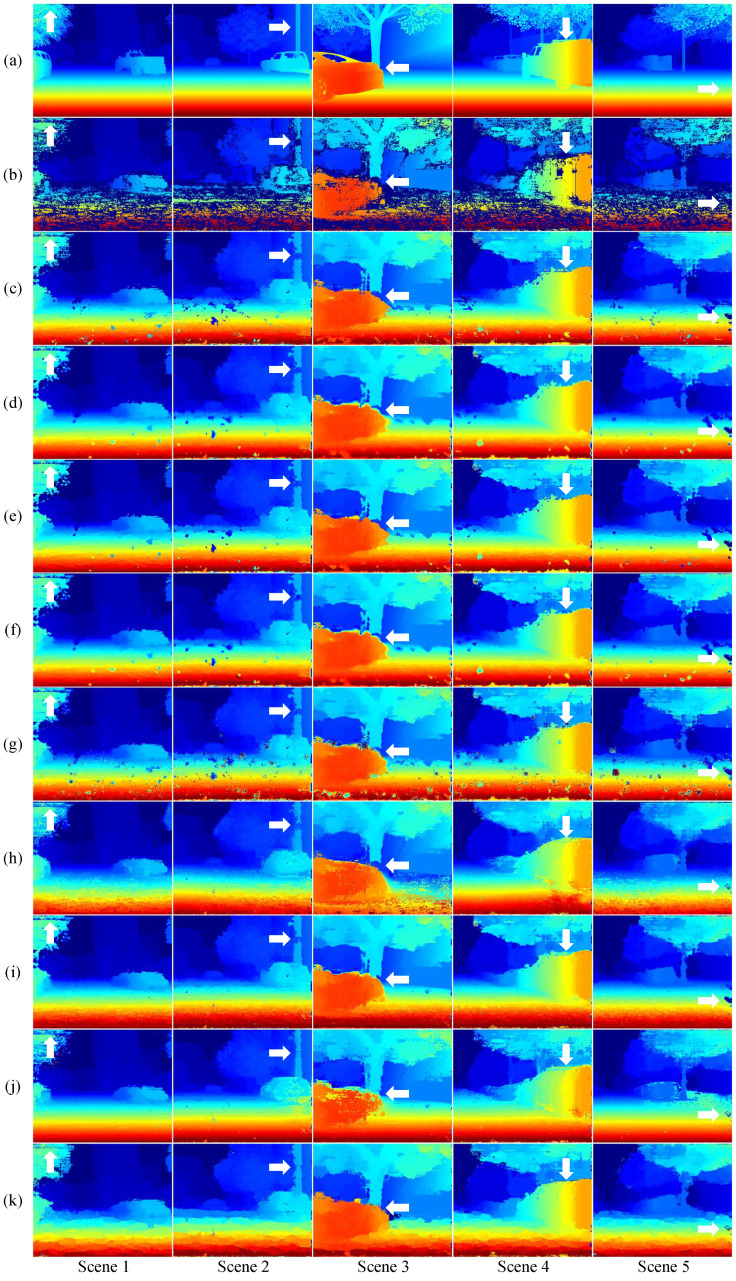
Disparity map comparisons on the synthetic Driving dataset across different scenes. (**a**) Ground truth, (**b**) SGM$\left(U_{th}=10\right)$, (**c**) SGM$\left(U_{th}=0\right)$, (**d**) Linear Interpolation, (**e**) Nearest Interpolation, (**f**) PDE, (**g**) ShCNN [56], (**h**) GMCNN [55], (**i**) MADF [58], (**j**) Chen [57], and (**k**) The proposed. Invalid regions are shown in darkish blue.

Firstly, the Sceneflow dataset [65], a large-scale synthetic dataset designed for depth estimation, consists of three subsets: FlyingThings3D, Driving, and Monkaa. For this study, we focused on the Driving dataset, which is specifically tailored to autonomous driving scenarios. Figure 7a shows the ground truth disparity maps from the dataset. To generate SGM disparity maps from the left and right images of the Driving dataset, we adjusted the “UniquenessThreshold” parameter, $U_{th}$ in the SGM function in MATLAB R2023b. Figure 7b presents the resulting disparity maps with $U_{th}=10$, which ensures that the disparities of the valid pixels closely match the ground truth while maximizing the proportion of the valid regions. However, the SGM disparity map still contains some invalid pixels, shown in dark blue, especially on the left sides of objects and in textureless regions. Figure 7c displays the SGM disparity maps with $U_{th}=0$, where no invalid pixels are generated, but a large portion of the disparities is incorrectly estimated. Consequently, the maps in Figure 7b with $U_{th}=10$ are considered the most reliable outcomes and are used as input images for comparing the performance of different methods. We compare the proposed method against basic interpolation methods, a PDE-based method, and four neural network-based methods.

The results of linear interpolation and nearest-neighbor interpolation are shown in Figure 7d,e. In these traditional interpolation methods, the pixels marked as invalid were masked and replaced with “NaN” to estimate the disparity. In the inpainting methods, which aim to identify invalid regions by masking them and restoring the missing areas, the masked regions were replaced with either the median disparity value or “NaN” to minimize errors caused by incorrect disparity estimation. Figure 7f,g,h,i,j show the results produced by the PDE-based inpainting method and the neural network-based methods, ShCNN [56] and GMCNN [55], MADF [58], Chen [57], respectively. ShCNN used a pre-trained model, while GMCNN, MADF, and Chen were directly trained using the ground truth from the Driving dataset. For GMCNN and Chen’s method, the mask is randomly generated according to the original code, while for MADF, the invalid pixels in the SGM output are used as the mask. The proposed method improves upon the SGM disparity map by using it as a prior and interpolating invalid pixels without introducing incorrect estimations, as illustrated in Figure 7k.

As shown in Figure 7b, a large number of invalid pixels occur in textureless areas, such as the floor, forming a large region. While the proposed method maintains consistent accuracy even in the large invalid regions, the basic interpolation methods and ShCNN tend to estimate incorrect disparity values as the size of the invalid region increases. These methods perform poorly in the lower half of each disparity map in Figure 7, where the proportion of invalid pixels is substantial. Although GMCNN was trained on data with similar scenes and shows sharper details in the upper half of the image, it still struggles with large invalid regions at the bottom, as seen in Figure 7g. MADF fills missing areas with fairly plausible values; however, as observed in the arrow-marked regions in Figure 7h, unexpected empty regions appear, a common issue in deep learning-based methods. Chen’s method, while producing smooth results in regular and repetitive lower regions such as roads, tends to estimate incorrect disparity values in the upper regions, which are rich in structure and non-repetitive patterns, as demonstrated in Figure 7j. In contrast, the proposed method uses the SGM prior to accurately fill invalid regions, preserving the integrity of the original valid data—something that existing methods generally fail to achieve. Notably, the proposed method does not smooth out the valid pixels in the SGM output.

To quantitatively measure the performance of each method, the Endpoint Error (EPE) is used as follows:

(5) $$\mathrm{EPE}=\frac{1}{\left|V\right|}\sum_{i\in V}\left|{\hat{d}}_{i}-d_{i}^{*}\right|$$

where *V* and |V| are the set of valid pixels and the number of valid pixels, respectively. ${\hat{d}}_{i}$ is the estimated disparity value for pixel *i*, and $d_{i}^{*}$ is the ground truth disparity value for pixel *i*. A summary of the endpoint errors and invalid pixel ratios for the various methods is provided in Table 1. The default SGM method shown in Figure 7b achieved the lowest EPE but marked only about 63 percent of all pixels in the dataset as valid, on average. Reducing $U_{th}$ to 0 increased the error to 7.77, setting this EPE as the baseline for further enhancement. The proposed method did not achieve the lowest overall EPE in synthetic datasets, primarily due to the regularity of road regions, which favor deep learning-based methods. To analyze this further, we evaluated the error separately for the upper (structure-rich) and lower (road) regions of the images, divided approximately in a 50:50 ratio. While deep learning methods performed better in the lower regions with repetitive patterns, our method showed improved accuracy in the upper regions containing complex structures and objects. This underscores the robustness of our approach in handling irregular and non-repetitive patterns, where learning-based methods often struggle.

### 4.2. Self-Generated Real-World Dataset

To evaluate the performance of the proposed method in enhancing ground truth SGM images used for training a neural network in disparity estimation for autonomous driving, we developed a comprehensive real-world dataset using a stereoscopic camera. In real-world scenarios, unlike the proposed method, deep neural networks often struggle to obtain ground truth. Therefore, their performance is evaluated by comparing how well a neural network trained on a specific dataset performs on unseen datasets. The self-generated dataset consists of synchronized left, right, and RGB images along with IR patterns to enhance the matching algorithm. A total of 4777 image pairs with the size of 848×480 were captured, covering distances ranging from 20 cm to infinity in various autonomous robotic environments.

#### 4.2.1. Ground Truth Disparity Map Comparison for Training Neural-Nets

**Figure 8:**
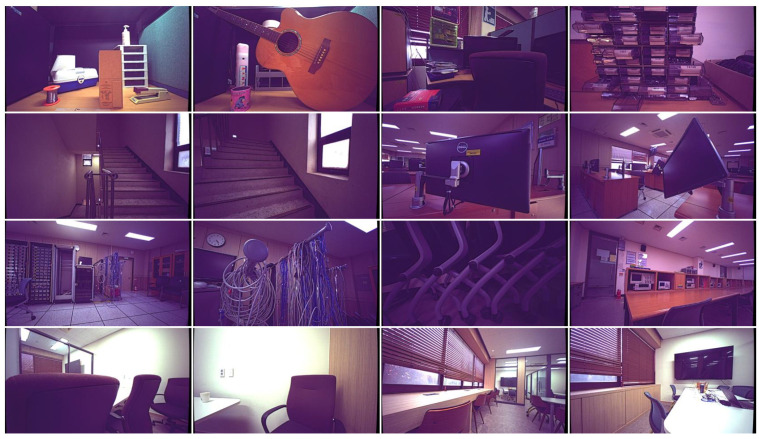
Example captured images of real-world indoor scenes.

**Figure 9:**
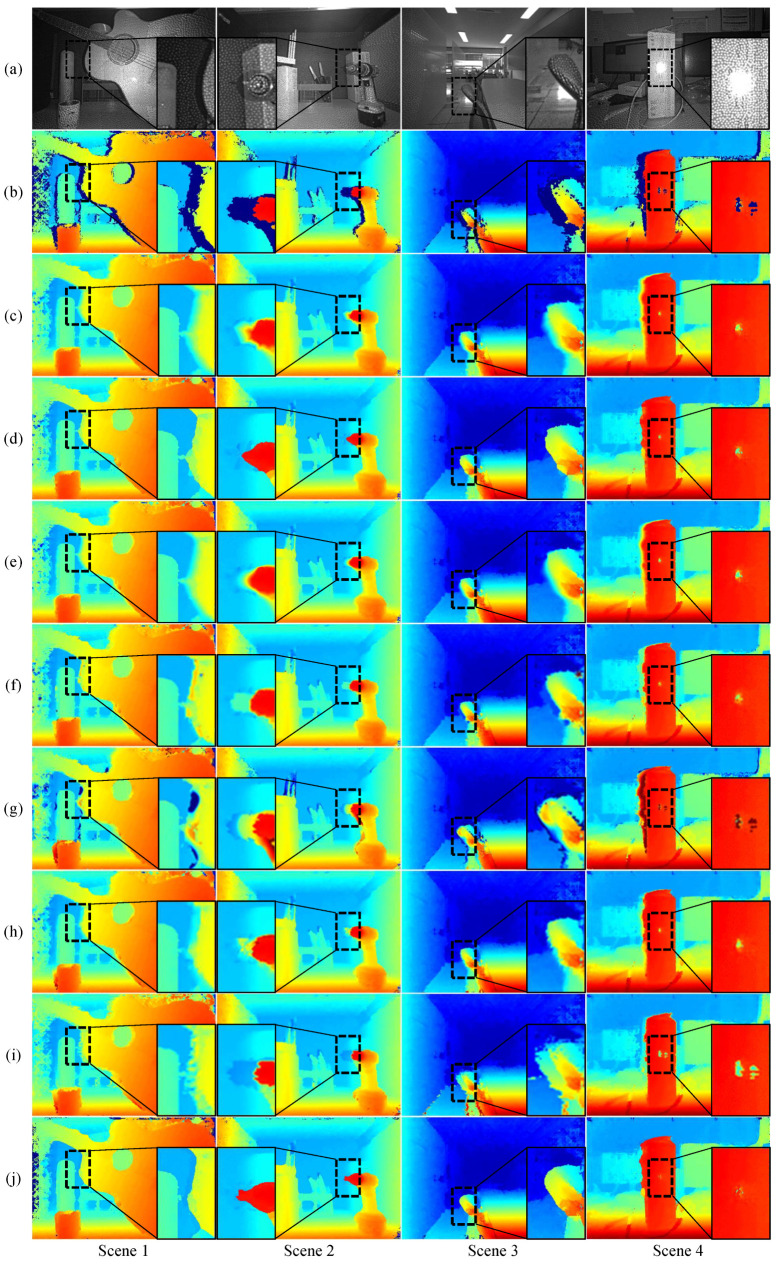
Disparity map comparisons across different real-world scenes. (**a**) Input left images, (**b**) SGM$\left(U_{th}=10\right)$, (**c**) Linear Interpolation, (**d**) Nearest Interpolation, (**e**) PDE, (**f**) Shepard inpainting [56], (**g**) GMCNN [55], (**h**) MADF [58], (**i**) Chen [57], and (**j**) The proposed. The insets highlight areas with significant differences, particularly in challenging regions with occlusions and textureless surfaces. Invalid regions are shown in darkish blue.

Figure 8 presents examples of the RGB images from the captured scenes. We first generated disparity maps using SGM and then applied the proposed interpolation method to these maps. Figure 9a shows the images captured by the left camera. As illustrated in Figure 9b, the initial SGM disparity maps contain invalid pixels, indicated by a dark blue color, particularly on the left side of objects. The presence of these invalid pixels is reduced compared to the synthetic dataset due to the IR patterns that create comparable features on flat surfaces. Unlike existing methods, the enhanced disparity maps produced by the proposed method clearly define object boundaries, as highlighted in the marked areas of the first to third columns in Figure 9. In the fourth column, it is evident that SGM produces incorrect disparities in the center of the marked rectangle along with invalid pixels. This error propagates through the interpolation methods, resulting in a significant amount of incorrect disparity, as shown in the comparison methods.

While deep learning-based comparison methods showed numerically superior performance on the synthetic dataset, they performed significantly worse than the proposed method in terms of subjective quality on unseen real-world data, which differs from the synthetic data. In real-world scenarios, where obtaining GT is challenging, neural network-based inpainting techniques struggle to achieve reliable interpolation performance. In contrast, the proposed method demonstrates comparable performance on real-world data to that observed on synthetic datasets, highlighting its ability to generalize effectively across diverse datasets, even under challenging real-world conditions.

#### 4.2.2. Neural Network Output Map Comparison

To evaluate how the enhanced disparity maps influence the training of neural networks when used as GT, we conducted extensive experiments with neural network models under two different scenarios using a self-generated real-world dataset. In the first scenario, the models were trained on the original disparity maps produced by SGM, serving as a baseline for performance comparison. These maps contain invalid pixels, particularly on the left side of objects. In the second scenario, the models were trained on the enhanced maps generated by the proposed interpolation method, designed to improve the quality and accuracy of the disparity maps. This dual-training approach enabled a thorough evaluation of the proposed interpolation method’s impact on the overall performance of the neural network models, particularly their ability to produce visually appealing and accurate results in the interpolated regions.

**Figure 10:**
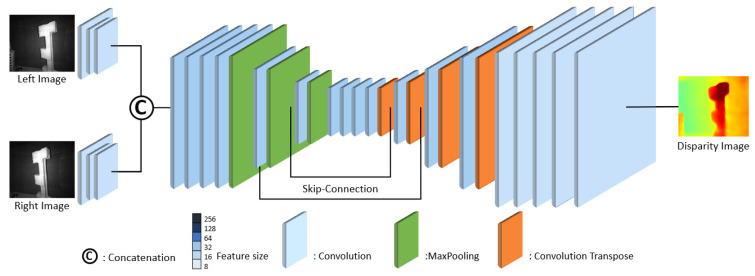
Basic CNN-based model for disparity estimation.

To this end, we compared three lightweight neural network models, each optimized for on-device AI, with PSMNet [50], a state-of-the-art disparity regression model. The first model in Figure 10 features a simple Encoder–Decoder architecture featuring Convolutional Layers with 16–32 features, Pooling Layers, and Convolutional Transpose Layers for upsampling. The Encoder reduces the input image dimensions through Convolutional and Pooling Layers, extracting key features. The Decoder then upsamples these features to restore the image to its original size, ultimately generating a pixel-wise disparity map. This basic neural network model includes approximately 220.93 K parameters and requires 21.8 GFLOPs to achieve real-time 30fps operation, which is more efficient compared to the 28.6 GFLOPs needed by YoloV8s. All FLOPs are measured based on an image size of 640×480.

**Figure 11:**
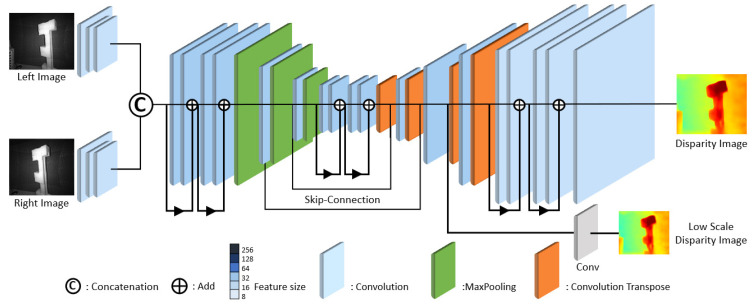
ResNet-based model for disparity estimation. Note that the additional low-scale intermediate feature maps are used to capture structural information of disparity at the original size.

The second model in Figure 11 incorporates a residual learning algorithm [66] to the Convolutional Layers to enhance the edges in invalid areas of the ground truth. There are no scores assigned to the invalid pixels in the training of the neural network, which makes the left-sided region of the object unclear. Rather than assigning pixel-by-pixel scores at the original image size, the second model employs low-scale intermediate features to capture structural information of disparity of the original size [65]. Therefore, low-scale disparity feature maps in the intermediate layer are extracted, convolved to reduce one channel disparity map, and additional loss at this scale is attached to the main loss. The model is designed with approximately 202.87 K parameters and requires 21.7 GFLOPs for computation.

**Figure 12:**
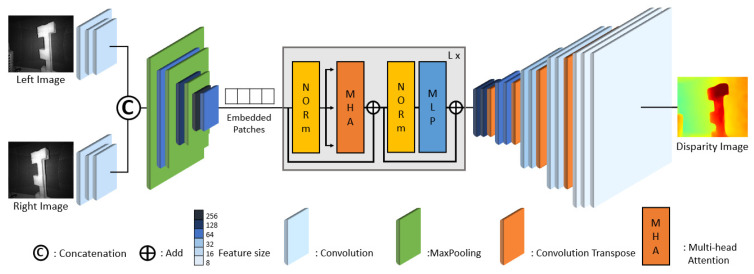
Vision Transformer-based model for disparity estimation. This model replaces the Encoder of the baseline model with a Vision Transformer (ViT) and modifies the Decoder from Figure 10 accordingly.

The third model replaces the Encoder of the baseline model with a Vision Transformer (ViT) [67] and modifies the Decoder accordingly. Figure 12 illustrates the structure of this third model. This neural network model is designed with approximately 1.79 M parameters and requires 14.9 GFLOPs for computation.

For comparison, we also evaluate these models against PSMNet. While PSMNet, with its 5.22 million parameters and 893.8 GFLOPs, is not feasible for onboard processing, it provides a useful benchmark for assessing the performance of the three models. Table 2 outlines the computational requirements for each neural network architecture.

A total of 4299 images were used for training, while an additional 478 images were set aside for testing. All models employed the L1 loss function as specified in Equation (6), with a batch size of 64 and a learning rate of 0.001, optimized using the Adam optimizer with exponential decay. During training, images were randomly cropped to 256×256 pixels, and intensity levels were scaled within the range from 0.5 to 1.5. To exclude invalid regions, a mask, as described in Equation (6), was applied during loss calculation. Additionally, the maximum disparity $d_{max}$ was set to 64 to cover the effective range of the camera.

(6) $$Loss=\sum_{i\in V}\left|{\hat{d}}_{i}-\min\left(d_{\max},d_{i}^{*}\right)\right|$$

**Figure 13:**
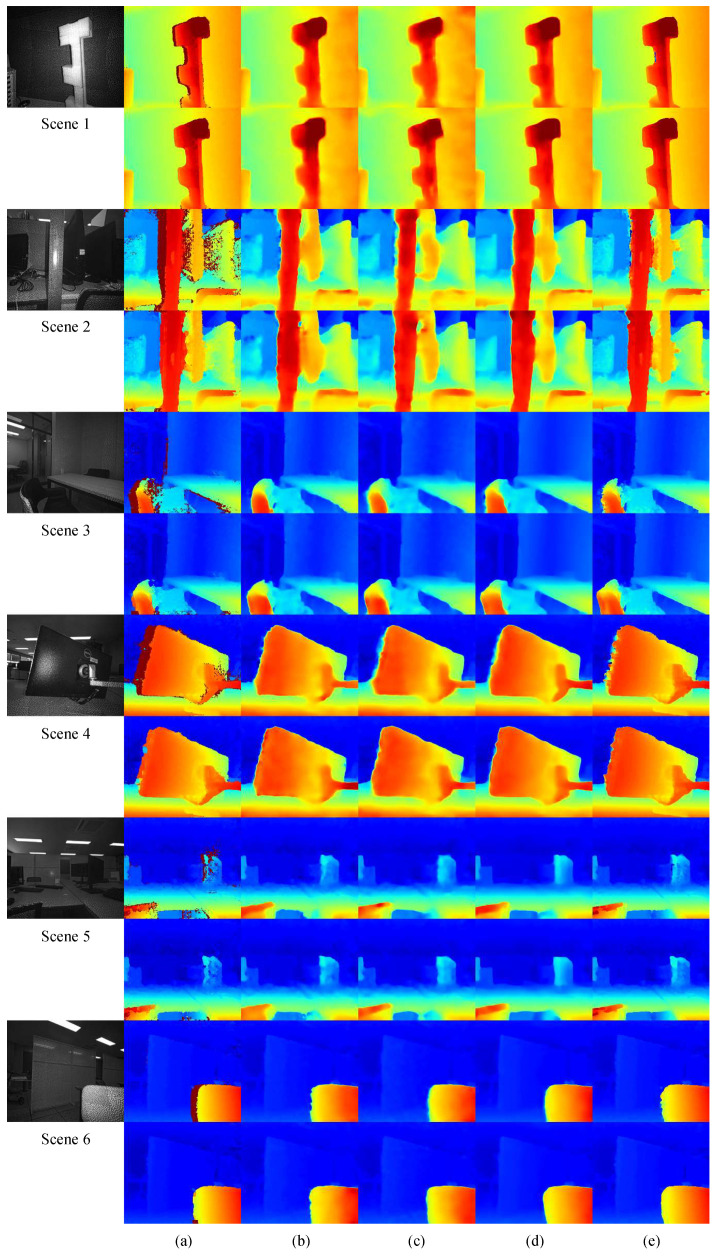
Disparity map comparisons across various scenes using different models. (**a**) GT, (**b**) CNN-based, (**c**) ResNet-based, (**d**) ViT-based, (**e**) PSMNet [50]. The first row of each scene is trained with the original SGM GT, and the second row with the proposed GT. Invalid regions are shown in darkish red.

The disparity maps presented in Figure 13 illustrate the outcomes from different neural network architectures. The first and second rows of each scene in Figure 13 represent models trained with SGM GT and the proposed GT, respectively. Across all architectures, the original SGM GT produces disparity maps with unclear edges, particularly along the left boundaries of objects where the ground truth was uncertain due to occlusions. In contrast, the proposed GT generates outputs with improved clarity, displaying slightly thicker but sharp boundaries as seen in the second row of the neural network models. PSMNet does produce more accurate disparities compared to the more concise models, the presence of invalid pixels results in a less organized overall disparity map, faithfully reproducing the unstructured details of SGM. The proposed method, by filling in the holes, creates cleaner and more organized boundaries.

Table 3 presents a comparison of endpoint errors across different neural network architectures. PSMNet achieved the lowest endpoint errors, followed by the transformer-based model, both when trained with SGM GT and the proposed GT. In the ResNet-based architecture, the proposed GT not only delivered visually improved disparity map but also a numerically superior disparity map compared to the original SGM GT.

## 5. Discussion

### Parameter Selection for Proposed Method

In the proposed MAP-based disparity estimation, there are controllable parameters: the window size $S_{w}$ for the prior, the patch size $S_{p}$ for the likelihood, and the intensity threshold $I_{th}$ for masking. To determine the optimal combination of these parameters for minimizing error while also reducing invalid regions, we randomly selected 400 images from the Driving dataset. Given the numerous possible combinations, we prioritized $S_{w}$ and $S_{p}$ in the initial analysis. Disparity maps were generated using the proposed method, varying $S_{w}$ and $S_{p}$ while keeping $I_{th}$ fixed.

**Figure 14:**
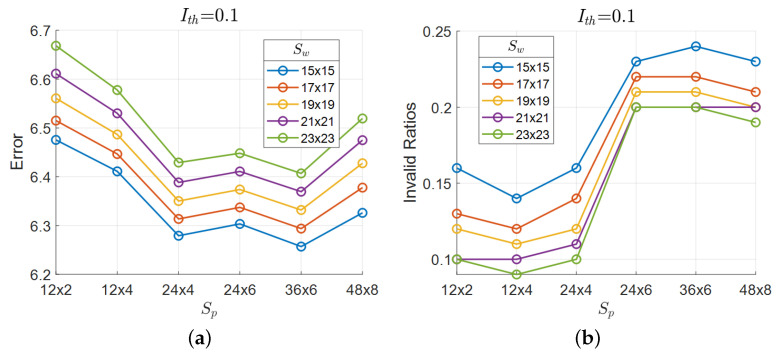
Relationship between patch size $S_{p}$ and both error and invalid pixel ratios for various prior window sizes $S_{w}$ at a fixed intensity threshold $I_{th}=0.1$. (**a**) shows how smaller values of $S_{w}$ and larger values of $S_{p}$ tend to minimize error, with an optimal configuration observed around $S_{w}=17\times 17$ and $S_{p}=24\times 4$. (**b**) demonstrates that smaller values of $S_{w}$ generally lead to higher invalid pixel ratios, while smaller values of $S_{p}$ help in reducing invalid pixel ratios. This indicates a trade-off in parameter selection between minimizing error and reducing invalid pixel ratios.

**Figure 15:**
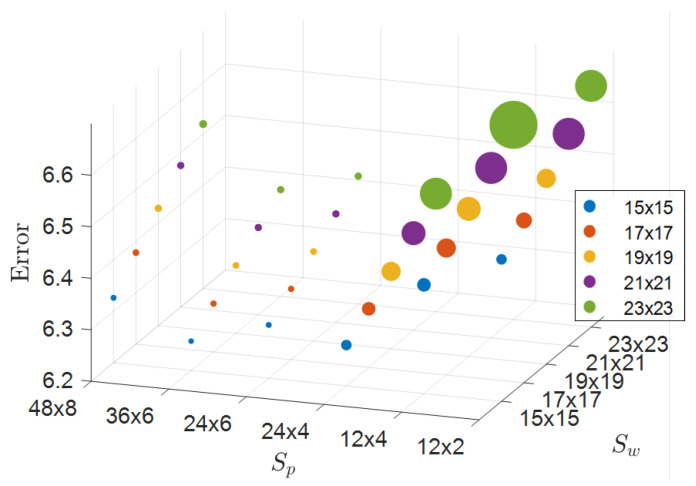
3D visualization of error-based on prior window size $S_{w}$ and patch size $S_{p}$, with point size indicating the reciprocal of invalid pixel ratios.

Figure 14a,b show results for $I_{th}=0.1$. From Figure 14a, the combination of a 15×15 window for $S_{w}$ and a 36×6 patch for $S_{p}$ yielded the lowest error. However, as shown in Figure 14b, smaller $S_{w}$ and larger $S_{p}$ tended to produce more invalid regions. Therefore, selecting parameters requires balancing the goals of minimizing error and reducing invalid regions. After evaluating these factors, we chose 17×17 for $S_{w}$ and 24×4 for $S_{p}$. Similar results were observed for other values of $I_{th}$. Figure 15 illustrates the relationship between these parameters and error in 3D space, with the intensity threshold held constant. In the plot, point size represents the reciprocal of the invalid pixel ratios, with larger points indicating smaller invalid pixel ratios. Generally, a larger $S_{p}$ is preferable, while a smaller $S_{w}$ yields better results. Consequently, we selected a 17×17 window for $S_{w}$ and a 24×4 patch for $S_{p}$ to balance error reduction and invalid pixel ratio minimization. Finally, with $S_{w}$ and $S_{p}$ fixed, we tested intensity thresholds among 10 different values, including “None,” and determined $I_{th}=-0.7$ as optimal.

## 6. Conclusions and Future Work

In this paper, we introduced a novel interpolation method to address invalid regions in SGM disparity maps using MAP estimation. By leveraging SGM outputs as prior probabilities and incorporating cosine similarity from surrounding regions, our approach effectively enhances the visual quality of disparity maps, particularly in challenging areas with large invalid regions, while suppressing irrelevant outliers. Although the interpolation may not always achieve exact numerical accuracy, the visually coherent disparity maps produced by our method enhance the performance of neural network models by providing more realistic and consistent training data. Moreover, the experimental results demonstrate the robustness of the proposed method in enhancing disparity maps, making it highly applicable to real-world environments where ground truth is unavailable. For future work, we aim to expand our ground truth enhancement methods to accommodate a broader range of real-world scenarios and challenges. Additionally, we plan to explore more sophisticated neural network architectures and optimization strategies to further enhance the accuracy and efficiency of disparity estimation for autonomous and real-time applications.

## Figures and Tables

**Table 1:** End-point error by region and invalid pixel ratio comparisons.

	Metric	SGM$\left(U_{\mathrm{th}}=10\right)$	SGM$\left(U_{\mathrm{th}}=0\right)$	Linear	Nearest	PDE	ShCNN [56]	GMCNN [55]	MADF [58]	Chen [57]	Proposed
**EPE**	**Overall**	4.87	7.77	7.30	7.48	7.37	8.87	8.51	6.36	**4.08**	6.68
	**Upper**	2.69	3.38	3.31	3.34	3.31	3.53	3.37	3.29	3.38	**3.25**
	**Lower**	10.15	12.19	11.33	11.65	11.46	14.06	13.69	9.45	**4.79**	10.15
**Invalid(%)**		37.04	0.00	0.00	0.00	0.00	0.00	0.00	0.00	0.00	0.15

**Table 2:** Computation comparison of neural networks tested, with FLOPs calculated for an image size of 640×480.

	CNN-Based	ResNet-Based	ViT-Based	PSMNet [50]
**# parameters**	220.93 K	202.87 K	1.79 M	5.22 M
**FLOPs**	21.8 G	21.7 G	14.9 G	893.8 G

**Table 3:** End-point error comparisons.

	CNN-Based	ResNet-Based	ViT-Based	PSMNet [50]
**SGM GT**	1.83	2.13	1.74	1.26
**Proposed GT**	1.91	2.03	1.87	1.35

## Data Availability

The original contributions presented in the study are included in the article, further inquiries can be directed to the corresponding author.
